# Scalable diversification options delivers sustainable and nutritious food in Indo-Gangetic plains

**DOI:** 10.1038/s41598-022-18156-1

**Published:** 2022-08-23

**Authors:** M. K. Gora, Satish Kumar, H. S. Jat, S. K. Kakraliya, Madhu Choudhary, A. K. Dhaka, R. D. Jat, Manish Kakraliya, P. C. Sharma, M. L. Jat

**Affiliations:** 1 CCS Haryana Agricultural University, Hisar, Haryana 125004 India; 2 ICAR-Central Soil Salinity Research Institute, Karnal, Haryana 132001 India; 3 International Maize and Wheat Improvement Center (CIMMYT), NASC Complex, Pusa, New Delhi 110012 India; 4 Resilient Farm and Food Systems Program, International Crops Research Institute for the Semi-Arid Tropics (ICRISAT), Patancheruvu, India

**Keywords:** Climate change, Environmental impact, Plant ecology

## Abstract

Indo-Gangetic plains (IGP) of South Asia have supported bulk of human and bovine population in the region since ages, and a spectacular progress has been made in food production. However, malnutrition, diminishing total factor productivity, and natural resource degradation continue to plague this cereal-dominated region, which is also vulnerable to climate change. Addressing these challenges would require a transition towards diversifying cereal rotations with agroecological cropping systems. A study was, therefore, conducted at the experimental farm of ICAR-CSSRI, Karnal on crop diversification and sustainable intensification options using agro-ecological approaches such as Conservation Agriculture (CA) and diversified cropping systems to ensure food and nutritional security while sustaining the natural resources. On 2 years mean basis, CA-based cropping system management scenarios (mean of Sc2–Sc7) using diversified crop rotations; increased the system yield by 15.4%, net return by 28.7%, protein yield by 29.7%, while using 53.0% less irrigation water compared to conventional tillage (CT)-based rice–wheat system (Sc1). Maize-mustard-mungbean on permanent beds (PBs) (Sc4) recorded the highest productivity (+ 40.7%), profitability (+ 60.1%), and saved 81.8% irrigation water compared to Sc1 (11.8 Mg ha^−1^; 2190 USD ha^−1^; 2514 mm ha^−1^). Similarly, Sc5 (maize-wheat-mungbean on PBs) improved productivity (+ 32.2%), profitability (+ 57.4%) and saved irrigation water (75.5%) compared to Sc1. In terms of nutritional value, Sc5 was more balanced than other scenarios, and produced 43.8, 27.5 and 259.8% higher protein, carbohydrate and fat yields, respectively, compared to Sc1 (0.93, 8.55 and 0.14 Mg ha^−1^). Scenario 5 was able to meet the nutrient demand of 19, 23 and 32 additional persons ha^−1^ year^−1^ with respect to protein, carbohydrate and fat, respectively, compared to Sc1. The highest protein water productivity (~ 0.31 kg protein m^−3^ water) was recorded with CA-based soybean-wheat-mungbean (Sc6) system followed by maize-mustard-mungbean on PBs (Sc4) system (~ 0.29 kg protein m^−3^) and lowest under Sc1. Integration of short duration legume (mungbean) improved the system productivity by 17.2% and profitability by 32.1%, while triple gains in irrigation water productivity compared to CT-based systems. In western IGP, maize-wheat-mungbean on PBs was found most productive, profitable and nutritionally rich and efficient system compared to other systems. Therefore, diversification of water intensive cereal rotations with inclusion of legumes and CA-based management optimization can be potential option to ensure nutritious food for the dwelling communities and sustainability of natural resources in the region.

## Introduction

Sustainability of the intensive cereal (rice/wheat) production systems of South Asia has become a major concern owing to overexploitation of natural resources through continuous cultivation of rice–wheat (RW) system. It has resulted into more water extraction from groundwater aquifers, soil degradation by intensive tillage and imbalance fertilizer use and open field burning of crop residue^1–3^. The problems are getting worse in western Indo-Gangetic plains (IGP) where rice and wheat are cultivated with Government’s investments for almost free electricity and highly subsidized nitrogen fertilizers, resulting in further over-drafting of ground water and highly skewed use (towards nitrogen) of chemical fertilizers with low NUE. In India, among the cereals, rice and wheat are massively distributed through public distribution system (PDS) to the large chunk of population to ensure food security throughout the country. But, the country has also been spending large sum for the imports of pulses, oils and other nutrition food product. Pulses and oilseeds, the major sources of protein and oil, are essential for growth and development of human body. Increasing the production of major pulses and oilseeds are the Government’s priority to fight against malnutrition by supplying it through PDS at affordable prices. Soil degradation, indiscriminate use of groundwater, injudicious use of fertilizer coupled with climate change effects, will make it increasingly difficult to produce sufficient amounts of food in future, while at present domestic per capita supply of some nutrient-rich foods such as legumes, oils and coarse grains has declined^4,5^.

The soil organic carbon (SOC) contents of cultivated soils particularly in RW system has decreased in last few decades compared to uncultivated virgin soils^6^, owing to intensive tillage, removal/burning of crop residues, mining of soil nutrient reserves in intensive RW systems. This adds to the challenge of making farming more profitable, and sustainable for resilient production systems and future food and nutritional security^7^. Moreover, there still exist large management yield gaps ranging from 14–47%, 18–70% and 36–77% in wheat, rice and maize, respectively^8^. The significant yields gap is mainly due to water and nutrient management^9^. Conventional tillage (CT) based management practices coupled with non-judicious use and inappropriate management of water, nutrients and crop residues for a longer period in RW system of IGP deteriorated the soil physical, chemical and biological properties^10,11^.

Crop diversification with sustainable intensification has been recognized as an effective strategy for achieving the objectives of food and nutritional security, sustainable management of land and water resources, and sustainable agricultural development^12,13^. Crop diversification, one of the major components of sustainable intensification in agriculture, helps in profit maximization through reaping the gains of complementary and supplementary relationships^14^. The necessity for crop diversification in IGP arises on account of (i) halting the groundwater decline, (ii) arresting the degradation of natural resources and the environment, (iii) attaining the self-reliance in pulses and oil to reduce  foreign exchange, and (iv) to ensure food and nutritional security. Choudhary et al*.*^1^ highlighted that sustainability of crop production increases with increase in crop diversity that improves the nutrition diversity and climate change mitigations, and reduces inorganic fertilizer use in associations with legumes enhancing production or profitability^15^. Studies revealed the inefficiencies present in food production systems in terms of water and nutrient use, showing the possibility of integration of crops (oilseeds and pulses) with lower groundwater requirements and also enhancing production of calorie, fats and protein^16,17^. Sustainable intensification of cereal systems may not only improve crop, water and nutrition productivity but also improve the soil and the environment health^1,18,19^.

Despite significant improvements in productivity in recent decades in cereal systems, rates of malnutrition remain high in South Asia, with adverse impacts on human, soil and environment. Therefore, RW systems need to be re-designed to reduce the impact of crop production on the environment, and for ensuring sustainable  food and nutrition security^20^. Sustainability of the natural resource in RW ecologies can potentially be achieved by pursuing conservation agriculture (CA) based sustainable intensification using diversity of crops in rotations, which are more friendly and efficient in utilizing natural resources. Presently, limited information is available on diversified cereal systems to ensure production of nutritious food on a sustainable basis. Our aim was to evaluate the performance and response of scalable diversified cropping systems on productivity (crop and water), profitability and nutritional outcome of systems as alternatives to conventional rice–wheat system in the western IGP of South Asia.

## Results

### Weather

**Figure 1:**
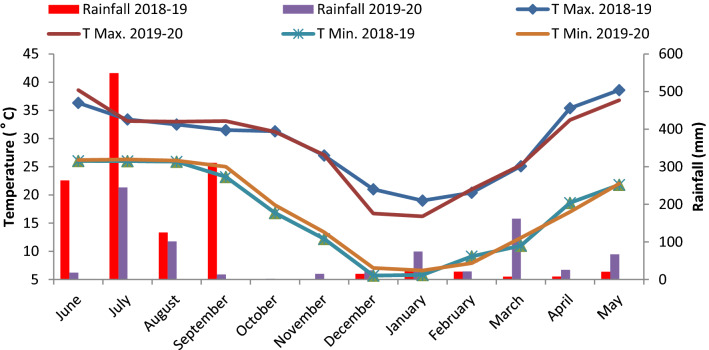
Yearly weather data of rainfall and temperature (max. and min.) for the year 2018–19 and 2019–20.

All the weather parameters measured during the study period are presented in Fig. 1. Crops received total rainfall of 1348 and 769 mm in 2018–19 and 2019–20, respectively, although it was not distributed uniformly through the season and the year. During first year *kharif* season, mostly rainfall was received in June-263 mm, July-549 mm, Aug-125 mm and Sep-311 mm, whereas in the second year it was 18, 245, 101 and 13 mm, respectively. During *rabi* season, 80 and 307 mm rainfall was received during both consecutive years. The maximum and minimum temperature was almost the same during both years. Daily maximum and minimum temperature were similar in both the year.

### Crops and system productivity

In this study, CA-based management practices significantly (p ≤ 0.05) influenced grain yields over the years (2018–2020) (Table 1). Rice yield was not significantly (p ≤ 0.05) influenced by the different rice based scenarios (Sc1–Sc3) in both the years. However, maize yield (rice equivalent; RE) in both the maize based scenarios (Sc4–Sc5) was significantly at par and higher by 36 and 46% (2-year mean) than rice crop of Sc1. Soybean yield (RE) under Sc5 was similar to Sc1 but lower than maize based scenarios in both the years. In contrast, pigeon pea (Sc7) produced significantly (*p* ≤ 0.05) lower yields than all scenarios in both the years. In Sc7, the RE yield of pigeon pea was lower by 30 and 60% during the first and second year, respectively compared to Sc1.

**Table 1:** Grain yield and net return of crops and cropping systems affected by different management practices during 2018–19 and 2019–20.

Scenarios^a^	Grain yield (Mg ha^−1^)				Net return (USD ha^−1^)			
	Rice/maize/ soybean/ pigeon pea	Wheat/ mustard	Mung bean	System^b^	Rice/maize/ soybean/ pigeon pea	Wheat/ mustard	Mungbean	System
**Year 2018–19**								
Sc1	5.73^Cc^	6.56^AB^	0.00	12.55^BC^	843^D^	1539^AB^	0.00	2382^B^
Sc2	6.00^BC^	7.03^A^	0.00	13.31^BC^	951^CD^	1666^A^	0.00	2617^B^
Sc3	5.58^C^	7.13^A^	0.18	13.70^B^	877^D^	1695^A^	3	2575^B^
Sc4	6.76^AB^ (7.04)^d^	6.16^BC^ (2.70)	0.71	15.98^A^	1388^AB^	1417^BC^	472	3277^A^
Sc5	7.51^A^ (7.82)	6.75^AB^	0.20	15.31^A^	1576^A^	1595^A^	50	3221^A^
Sc6	5.51^C^ (2.87)	5.73^C^	0.17	12.15^C^	1141^BC^	1320^C^	24	2486^B^
Sc7	4.01^D^ (1.25)	5.80^C^	0.63	12.54^BC^	809^D^	1343^C^	383	2535^B^
**Year 2019–20**								
Sc1	5.95^BC^	4.87^B^	0.00	11.06^D^	823^C^	1175C	0.00	1998^D^
Sc2	5.77^BC^	5.69^A^	0.00	11.75^D^	756^C^	1520AB	0.00	2276^C^
Sc3	5.42^C^	5.51^A^	0.00	11.20^D^	700^C^	1454B	-131	2023^D^
Sc4	9.16^A^ (9.55)	4.81^B^ (2.09)	0.77	17.24^A^	2071^A^	1122C	542	3735^A^
Sc5	9.50^A^ (9.90)	5.50^A^	0.17	15.92^B^	2159^A^	1487AB	30	3675^A^
Sc6	6.12^B^ (3.03)	5.98^A^	0.14	12.93^C^	1301^B^	1635A	9	2939^B^
Sc7	2.38^D^ (0.75)	5.73^A^	0.75	11.35^D^	400^D^	1565AB	493	2457^C^

^a^Refer Table 4 for description of scenarios.

^b^System grain yield was expressed as rice-equivalent yield (Mg ha^−1^).

^c^Means followed by a similar uppercase letter within a column are not significantly different at 0.05 level of probability using Tukey’s HSD test.

^d^Values in parenthesis indicate the actual yield of crop.

During the first year, higher wheat yield was recorded with Sc2, Sc3 and Sc5 compared to other scenarios, whereas, in second year, almost similar wheat yield was recorded under all the wheat based scenarios except Sc1 and Sc4. The wheat yield under ZT flat system (Sc2 and Sc3) was 10.9% (2-year mean) higher than Sc1 (farmers’ practice). Whereas, under permanent beds system (PBs; Sc5 to Sc7), it was only 3.5% higher. In the first year, higher wheat yield was recorded with ZT flat system compared to PB system, whereas in second year it was reversed (Table 1). Wheat equivalent yield under Sc4 (mustard on PB) was similar to CT-based scenario (Sc1) during both the years. CA-based mungbean scenarios produced 0.09, 0.74, 0.18, 0.15 and 0.69 Mg ha^−1^ (2-year mean) additional mungbean yield under Sc3, Sc4, Sc5, Sc6 and Sc7, respectively, compared to Sc1 where mungbean crop was not taken (Table 1). Among the scenarios, mungbean yield under Sc4 (maize based) produced highest yield closely followed by and Sc7 (pigeon pea based).

System yield (RE) varied from 11.06 to 17.24 Mg ha^−1^ during both the years of study (Table 1). On 2-year mean basis, Sc4 followed by Sc5 recorded higher yield over Sc1during both years. However, the lowest system yield was recorded with Sc7 (11.95 Mg ha^−1^) (Table 1). On 2 years mean basis, system yield (RE) of CA-based scenarios (Sc2–Sc7) was increased by ~ 15% i.e. 1.81 Mg ha^−1^ over the CT-based scenario (11.81 Mg ha^−1^). The system yield under ZT flat system (Sc2 and Sc3) was 5.8% (2-year mean) higher than Sc1 (farmers’ practice). However, under PBs (Sc4 to Sc7) it was improved by 20.1%. CA-based, crop diversification of CT based rice–wheat system with maize-wheat/mustard-mungbean increased the system yield by ~ 37% (2-year mean). Rice (Sc2–Sc3) and maize (Sc4–Sc5) based systems recorded ~ 6 and 37% (2-year mean) higher system productivity. The contrast effects were significant to system productivity associated with different CA-based management practices (Table 3).

### Economic profitability

The cultivation cost mainly attributed to field preparation, crop establishment, irrigation, fertilizer, pest management, harvesting/threshing, and man-days involved in crop production. The net return of rice, maize, soybean and pigeon pea varied from 809 to 1576 USD ha^−1^ in first year and 400–2159 USD ha^−1^ during second year (Table 1). Net returns under scenario 5 increased by 124.2% followed by Sc4 (107.6%) compared to Sc1 (833 USD ha^−1^). Whereas, the lowest (-27.4% from Sc1) was recorded with pigeon pea crop in Sc7 (605 USD ha^−1^). Rice crop (Sc2-Sc3) recorded almost similar net returns to farmers’ practice, whereas maize crop increased the net return by 115.9%.

The net returns in the *rabi* season varied from 1320 to 1695 USD ha^−1^ during both the years (Table 3). The higher net returns were recorded with Sc2, Sc3 and Sc5 by 17.4, 16.0 and 13.6% compared to Sc1 (1357 USD ha^−1^), respectively. The wheat net returns under ZT flat system (Sc2 and Sc3) were 16.7% (2-year mean) higher than Sc1 (farmers’ practice), however under PBs (Sc5 to Sc7) it was higher by 9.9% only. On 2 years mean basis, mungbean crop produced the net returns in order of Sc4 > Sc7 > Sc5 > Sc6. Whereas, the negative returns (−64 USD ha^−1^) were recorded under Sc3 (Table 1).

The system net returns varied from 1998 to 3735 USD ha^−1^ under different management practices over the years (Table 1). Scenario 4 recorded 37.6 and 86.9% net returns over Sc1 during first and second year, respectively, and it was closely followed by Sc5 (35.2 and 83.9%). However, the lowest net returns were recorded with Sc3 as compared to Sc1. System net returns of CA-based scenarios (Sc2–Sc7) were increased by 28.7% (2-year mean), and is equivalent to 628 USD ha^−1^ over the CT-based scenario (2190 USD ha^−1^). The system yield under ZT flat system (Sc2 and Sc3) was 8.3% (2-year mean) higher than Sc1 (farmers’ practice), however under PBs (Sc4 to Sc7) it was higher by 38.8%. CA-based, crop diversification of CT based rice–wheat system with maize-wheat/mustard-mungbean increased the system net return by 58.8% (2-year mean).

### Irrigation water use and water productivity

**Figure 2:**
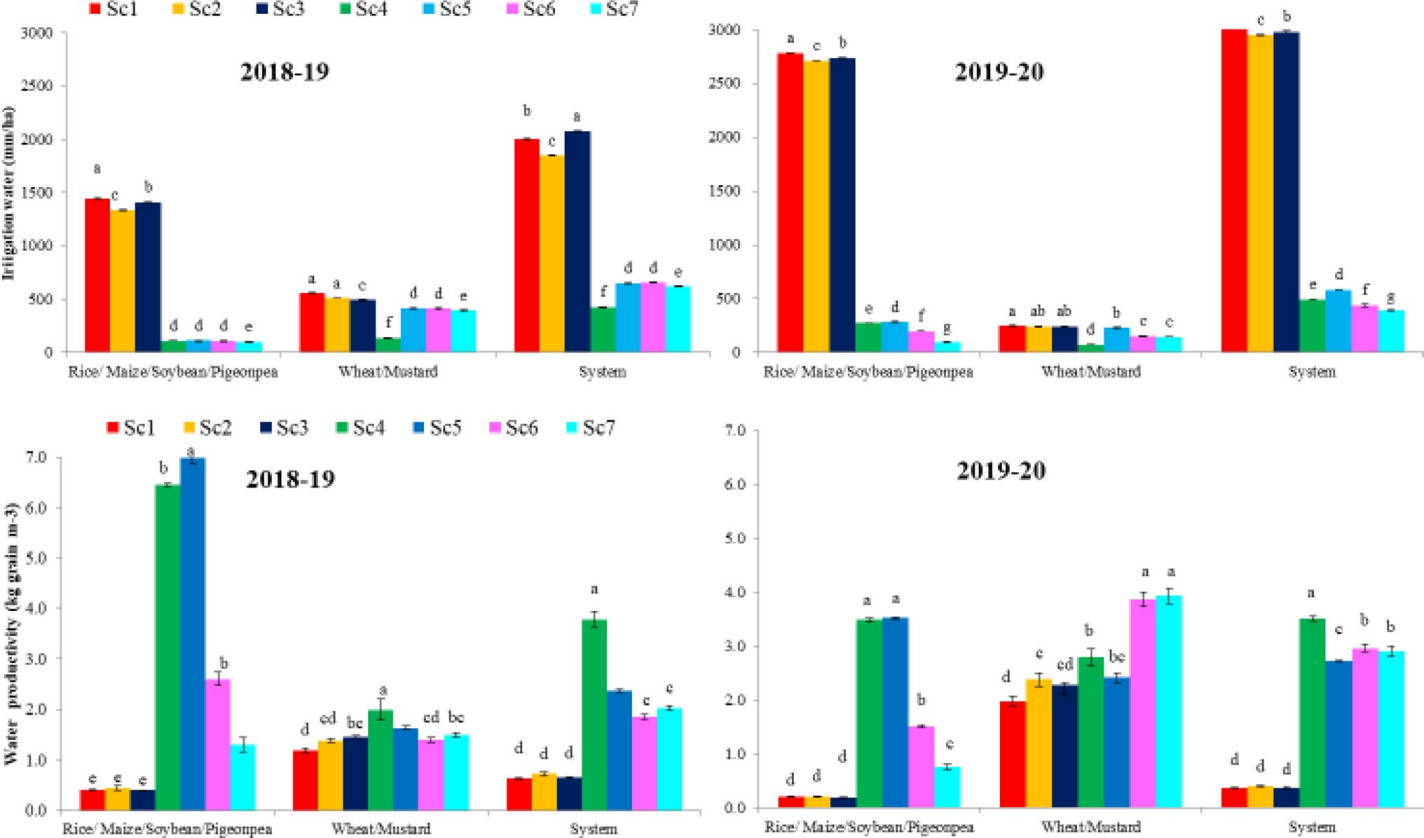
Irrigation water use (mm ha^−1^) and water productivity (kg grain m^−3^) as affected by different cropping systems under various management scenarios (2 years mean).

Different crops need variable amount of irrigation water to meet their evapotranspiration demand. The amount of irrigation water applied varied from 1336 to 2782 mm ha^−1^ in rice, 109 to 281 mm ha^−1^ in maize, 110–200 mm ha^−1^ in soybean and 96–100 mm ha^−1^ in pigeon pea over the 2-years. The amount of water applied in CT-based rice crop (Sc1; farmers’ practice) was significantly (*P* < 0.05) higher by ~ 3, 91, 93 and 95% (2-years’ mean) compared to CA-based rice (Sc2–Sc3), maize (Sc4–Sc5), soybean (Sc6) and pigeon pea (Sc7) scenarios, respectively (Fig. 2). However, diversified crops (maize, soybean and pigeon pea; Sc4-Sc7) saved ~ 92% of irrigation water compared to Sc1 (2114 mm ha^−1^) (2 years mean). The lowest irrigation water productivity (WP_I_) was recorded with rice and it ranged from 0.21 to 0.45 kg grain m^−3^. Highest WP_I_ was observed with maize crop and it ranged from 3.49 to 6.99 kg grain m^−3^ across the years (Fig. 2). However, in soybean and pigeon pea, it was recorded from 1.51–2.61and 0.75–1.30 kg grain m^−3^.

Crop establishment influenced the water use significantly in wheat crop. The irrigation water use in wheat crop ranged from 146 to 555 mm ha^−1^ across the years, while in mustard (Sc4) it was 73–136 mm ha^−1^. The wheat irrigation water use under PBs (Sc5 to Sc7) was 27.5% (2- year mean) lower than Sc1 (farmers’ practice), however under ZT flat system (Sc2 and Sc3) it was only 7.4% lower (Fig. 2). CA-based scenarios (mean of Sc2 to Sc7) saved 29.4 and 26.6% irrigation water during first and second year, respectively, compared to Sc1. The WP_I_ ranged from 1.18 to 3.94 kg grain m^−3^ during *rabi* season. Highest WP_I_ was recorded with Sc7 and Sc6 closely followed it during the study. CA-based scenarios (Sc2–Sc7) improved the WP_I_ by 72 and 31% during first and second year, respectively, compared to Sc1. CA-based mungbean scenarios used 87.0, 161.4, 97.5, 109.2 and 138.0 mm ha^−1^ (2-year mean) additional irrigation water under Sc3, Sc4, Sc5, Sc6 and Sc7, respectively, compared to Sc1 where mungbean crop was taken (Fig. 2).

System irrigation water use varied from 391 to 3027 mm ha^−1^ during both the years of study (Fig. 2). Scenario 4 recorded the 78.9 and 83.8% lower water use over Sc1 (2000 and 3027 mm ha^−1^) during first and second year, respectively, and it was followed by Sc7 (69.1 and 87.1%). On 2 years mean basis, the lowest water use was recorded with Sc4 (81.8%) followed by Sc7 (79.9%) compared to Sc1 (2514 mm ha^−1^). On 2 years mean basis, system irrigation water use of CA-based scenarios (Sc2–Sc7) was saved by ~ 53% i.e. 1339.5 mm ha^−1^over the CT-based scenario (2514 mm ha^−1^). The system irrigation water use under ZT flat system (Sc2 and Sc3) was 2.1% (2-year mean) lower than Sc1 (farmers’ practice), however under PBs (Sc4 to Sc7) it was saved by 78.9% with different cropping systems. Scenario 4 recorded 503.6 and 861.7% higher water productivity over Sc1 (0.63 and 0.37 kg grain m^−3^) during first and second year, respectively, followed by Sc5 (276.8 and 649.3%). On 2 years mean basis, CA-based management practices improved water productivity by 203.3 and 488.4% during first year and second year, respectively, as compared to farmers’ practice (Sc1). CA-based, crop diversification of CT based rice–wheat system with maize-wheat/mustard-mungbean increased the system water productivity by ~ 301% (2-year mean).

### Protein yield

In present study, different crops and their management practices significantly (*p* ≤ 0.05) influenced the protein yields (Table 2). Sc6, Sc5 and Sc4 improved the protein yield by 179.0, 63.2and 50.0% in first year and 145.0, 80.0 and 97.5% in second year, respectively, compared to Sc1 (0.38 and 0.40 Mg ha^−1^). However, Sc3 and Sc7 produced 3.8% and 46.2% lower protein yield than Sc1, respectively (Table 2). Based on 2-year mean, Sc6 (soybean on PBs) was recorded the highest protein yields of 1.02 Mg ha^−1^ which was 161.5% higher than Sc1 (0.39 Mg ha^−1^). Linear contrast effects were significant to protein yield of CT and CA-based scenarios in both the years (Table 3).

**Table 2:** Protein, carbohydrates and fat yield of different crops and cropping systems as affected by different management practices during the year 2018–19 and 2019–20.

Scenarios^a^	Protein yield (Mg ha^−1^)				Carbohydrates yield (Mg ha^−1^)				Fat yield (Mg ha^−1^)			
	Rice/maize/ soybean/ pigeon pea	Wheat/ mustard	Mungbean	System	Rice/maize/ soybean/ pigeon pea	Wheat/ mustard	Mungbean	System	Rice/maize/ soybean/ pigeon pea	Wheat/ mustard	Mungbean	System
**Year 2018–19**												
Sc1	0.38^Cb^	0.62^BC^	0.00	1.01^D^	4.50^BC^	4.59^B^	0.00	9.09^B^	0.03^C^	0.11^B^	0.00	0.15^D^
Sc2	0.40^C^	0.67^AB^	0.00	1.08^CD^	4.72^BC^	4.93^AB^	0.00	9.66^B^	0.04^C^	0.11^B^	0.00	0.14^D^
Sc3	0.38^C^	0.69^A^	0.04	1.11^C^	4.36^C^	5.01^A^	0.11	9.49^B^	0.03^C^	0.10^B^	0.00	0.14^D^
Sc4	0.57^B^	0.52^D^	0.17	1.25^B^	5.11^AB^	0.77^D^	0.44	6.32^C^	0.30^B^	1.06^A^	0.01	1.37^A^
Sc5	0.62^B^	0.68^AB^	0.05	1.35^B^	5.74^A^	4.72^AB^	0.12	10.58^A^	0.34^B^	0.12^B^	0.00	0.47^C^
Sc6	1.06^A^	0.57^CD^	0.04	1.66^A^	0.97^D^	3.96^C^	0.11	5.04^D^	0.56^A^	0.12^B^	0.00	0.68^B^
Sc7	0.26^D^	0.61^C^	0.15	1.02^CD^	0.83^D^	4.01^C^	0.40	5.24^D^	0.02^C^	0.12^B^	0.01	0.14^D^
**Year 2019–20**												
Sc1	0.40^C^	0.44^C^	0.00	0.84^C^	4.67^B^	3.33^B^	−0.00	8.01^B^	0.04^D^	0.09^B^	0.00	0.13^D^
Sc2	0.39^C^	0.52^AB^	0.00	0.91^C^	4.51^B^	3.93^A^	−0.00	8.44^B^	0.04^D^	0.10^B^	0.00	0.14^D^
Sc3	0.37^C^	0.51^B^	0.00	0.87^C^	4.22^B^	3.79^A^	−0.00	8.02^B^	0.03^D^	0.09^B^	0.00	0.12^D^
Sc4	0.79^B^	0.39^C^	0.19	1.36^B^	7.09^A^	0.58^C^	0.48	8.16^B^	0.38^C^	0.82^A^	0.01	1.21^A^
Sc5	0.72^B^	0.55^AB^	0.04	1.31^B^	7.33^A^	3.80^A^	0.10	11.23^A^	0.43^B^	0.09^B^	0.00	0.52^C^
Sc6	0.98^A^	0.55^AB^	0.03	1.56^A^	1.04^C^	4.14^A^	0.08	5.26^C^	0.58^A^	0.09^B^	0.00	0.67^B^
Sc7	0.16^D^	0.58^A^	0.18	0.92^C^	0.50^D^	3.94^A^	0.47^A^	4.91^C^	0.01^D^	0.10^B^	0.01	0.12^D^

^a^Refer Table 4 for description of scenarios.

^b^Means followed by a similar uppercase letter within a column are not significantly different at 0.05 level of probability using Tukey’s HSD test.

**Table 3:** Significance effects of different agronomic management practices and their linear contrast on grain yield, net return, protein yield, carbohydrate yield and fate yield under different scenarios during 2018–19 and 2019–20.

Scenarios	Grain yield (Mg ha^-1^)			Net return (USD ha^-1^)			Protein yield (Mg ha^-1^)			Carbohydrates yield (Mg ha^-1^)			Fat yield (Mg ha^-1^)		
	Rice/maize/soybean/ pigeon pea	Wheat/mustard	System	Rice/maize/soybean/ pigeon pea	Wheat/mustard	System	Rice/maize/soybean/ pigeon pea	Wheat/mustard	System	Rice/maize/soybean/ pigeon pea	Wheat/mustard	System	Rice/maize/soybean/ pigeon pea	Wheat/mustard	System
**Year 2018–19**															
CT vs CA	NS	NS	NS	*	NS	NS	**	NS	**	**	***	***	***	***	***
RW vs MMs	NS	NS	***	***	NS	**	***	***	NS	*	***	***	***	***	***
RW vs MW	***	NS	***	***	NS	**	***	NS	***	***	NS	**	***	NS	***
RW vs SW	NS	***	NS	*	**	NS	***	NS	***	***	***	***	***	NS	***
RW vs PW	***	***	NS	NS	*	NS	**	NS	NS	***	***	***	NS	NS	NS
MM vs MW	NS	NS	NS	NS	*	NS	NS	***	*	NS	***	***	NS	***	***
SW vs PW	*	NS	NS	*	NS	NS	***	NS	***	NS	NS	NS	***	NS	***
**Year 2019–20**															
CT vs CA	NS	**	***	***	**	***	***	*	***	***	NS	NS	***	***	***
RW vs MMs	***	*	***	***	***	***	***	**	***	***	***	NS	***	***	***
RW vs MW	***	NS	***	***	NS	***	***	NS	***	***	NS	***	***	NS	***
RW vs SW	NS	*	***	***	NS	***	***	NS	***	***	**	***	***	NS	***
RW vs PW	***	NS	NS	***	NS	NS	***	**	NS	NS	NS	***	NS	NS	NS
MMs vs MW	NS	*	**	NS	***	NS	NS	***	NS	NS	***	***	**	***	***

*CT* conventional tillage (Sc1), *CA* conservation agriculture (Sc2–Sc7), rice–wheat (Sc1–Sc3), *MMs* maize-mustard (Sc4), *MW* maize-wheat (Sc5), *SW* soybean-wheat, *PW* pigeon pea-wheat.

*Significance at the *p* < 0.05

**Significance at the *p* < 0.01

***Significance at the *p* < 0.001.

The protein yield varied from 0.44 to 0.69 Mg ha^−1^ in wheat crop and 0.39–0.52 in mustard crop during both the years (Table 2). On 2 years mean basis, 16.0% higher protein yield was recorded with Sc5, closely followed by Sc3 (13.2%) compared to Sc1 (0.53 Mg ha^−1^). CT (Sc1) *vs* CA based scenarios (mean of Sc2-Sc7) contrast effect was not significant on wheat protein yield in 2018–19, but it was significant in 2019–20. CA-based mungbean scenarios produced 0.02, 0.18, 0.05, 0.04 and 0.17 Mg ha^−1^ additional protein yield from mungbean under Sc3, Sc4, Sc5, Sc6 and Sc7, respectively, compared to Sc1 (0.0 Mg ha^−1^). Among the mungbean scenarios, Sc4 (mungbean on PBs) produced the highest protein yield compared to other mungbean scenarios (mean of 2 years).

System protein yield varied from 0.84 to 1.66 Mg ha^−1^ during both the years of study (Table 2). Scenario 6 recorded the 64.4 and 85.7% higher protein yield over Sc1 (1.01 and 0.84 Mg ha^−1^) during first and second year, respectively, followed by Sc5 (33.7 and 56.0%). However, the lowest (0.93 Mg ha^−1^) protein yield was recorded with Sc1. On 2 years mean basis, Sc6 and Sc5 recorded 74.1 and 43.9% higher protein yields over Sc1 (0.93 Mg ha^−1^). The percent increment of protein yields over Sc1 was in order of Sc6 > Sc5 > Sc4 > Sc2 > Sc3 > Sc7 on 2 years mean basis (Table 2). System protein yield (2-year mean) of CA-based scenarios (Sc2–Sc7) improved 29.7% compared to Sc1. The protein yield under ZT flat system (Sc2 and Sc3) was 7.3% (2-year mean) higher than Sc1 (farmers’ practice), however under PBs (Sc4 to Sc7) higher by 41.0%. The contrast effects were significant to system protein productivity associated with different CA-based management practices (Table 3).

### Carbohydrate yield

Crops and their management practices significantly (*p* ≤ 0.05) influenced the carbohydrate yield. In the first year, higher carbohydrate yield was recorded with Sc2, Sc4 and Sc5, whereas in second year it was recorded with Sc4 and Sc5 (Table 2). However, other scenarios i.e. Sc3, Sc6 and Sc7 produced the lower carbohydrate yield compared to Sc1 during both years. Maize crop produced the higher carbohydrate yield of 33.0 and 42.5% in Sc4 and Sc5 compared to Sc1 (4.59 Mg ha^−1^). However, soybean and pigeon pea produced 78.1 and 85.5% lower carbohydrate yield than Sc1, respectively (Table 2). Contrast effects (CT vs. CA) were significant to carbohydrate under different scenarios and combinations of management practices (Table 3).

The carbohydrate yield under wheat crop varied from 3.33 to 5.01 Mg ha^−1^ during both the years (Table 2). Carbohydrate yield (2-year mean) improved by 11.9 and 11.1% in Sc2 and Sc3, respectively compared to Sc1 (3.96 Mg ha^−1^). Contrast effects were significant to carbohydrate yield in first year and non-significant in second year. CA-based mungbean scenarios produced 0.05, 0.46, 0.11, 0.10 and 0.44 Mg ha^−1^ (2-year mean) additional carbohydrate yield under Sc3, Sc4, Sc5, Sc6 and Sc7, respectively, compared to farmers’ practice (0.0 Mg ha^−1^). Among the mungbean scenarios, CA-based Sc4 and Sc7 produced higher carbohydrate yield compared to other mungbean scenarios.

System carbohydrate yield varied from 4.91 to 11.23 Mg ha^−1^ during both the years (Table 2). Sc5 recorded the highest carbohydrate yield of 16.4% and 40.2% over Sc1 (9.09 and 8.01 Mg ha^−1^) during first and second year, respectively, followed by Sc2 (6.3 and 5.4%) and Sc3 (4.4 and 0.1%). However, the lowest (5.1 Mg ha^−1^) carbohydrate yield was recorded with Sc7 (pigeon pea based system). On 2 years mean basis, Sc5, Sc2 and Sc3 recorded 27.5, 5.8 and 2.4% higher carbohydrate yields over Sc1 (8.6 Mg ha^−1^). Contrast effects (CT *vs* CA) were significant to system carbohydrate yield in first year and non-significant in second year (Table 3).

### Fat yield

Fat yield under different scenarios in 2 years varied from 0.01 to 0.58 Mg ha^−1^ (Table 2). The fat yield was higher by 1528.6 and 1000% (2-year mean) with Sc6 and Sc5, respectively, compared to Sc1 (0.035 Mg ha^−1^). Fat yield was 871.4 and 14.3% higher with Sc4 and Sc2, respectively, as compared to Sc1. In both the years, contrast effects were significant to fat yield associated with different CA-based management practices (Table 3).

No appreciable change in fat yield was recorded under wheat crop, however, mustard crop produced the 863.6 and 811.1% higher fat yield in first and second years compared to Sc1 (wheat crop), respectively. In both the years, contrast effects were significant to system fat yield associated with different CA-based management practices (Table 3). Among the mungbean scenarios, CA-based Sc4 and Sc7 produced a little fat yield compared to other mungbean scenarios (Table 2).

System fat yield varied from 0.12 to 1.37 Mg ha^−1^ during both the years (Table 2). On 2 years mean basis, Sc4, Sc6 and Sc5 recorded 821.4, 382.1 and 253.6% higher fat yields over Sc1 (0.14 Mg ha^−1^). Compared to CT-based RW system, fat yield was lower by ~ 7.0% in both Sc3 and Sc7. The contrast effects were significant to system fat yield associated with different CA-based management practices (Table 3).

### Systems nutritional efficiency

**Figure 3:**
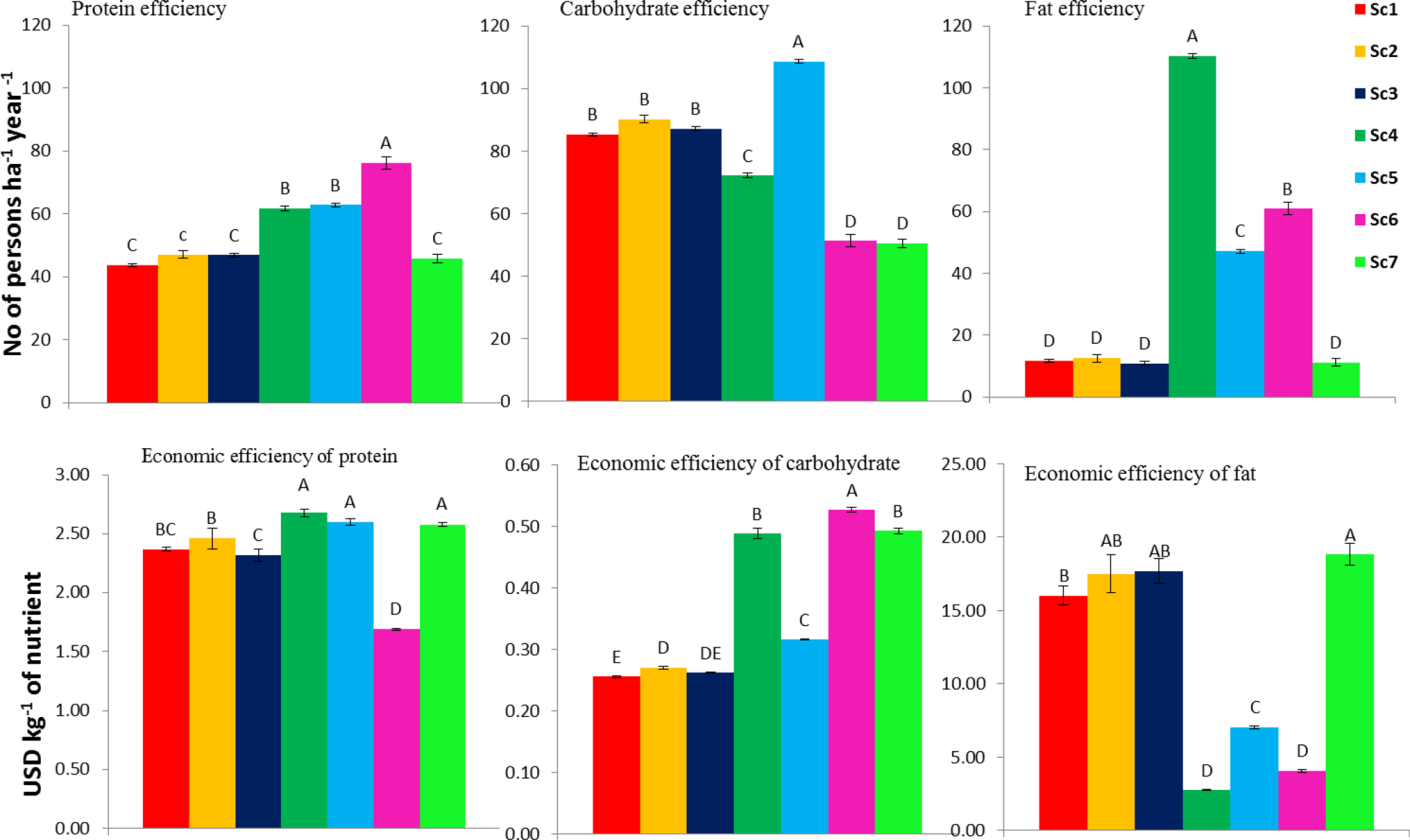
Yearly protein, carbohydrate and fat demand (based on 58, 275 and 30 g day^−1^ adult^−1^) equivalents for adults and economic efficiency of protein, carbohydrate and fat as affected by different cropping systems under various management scenarios (2 year`s mean).

On protein demand equivalent basis, CA-based scenarios could meet out the adult protein demand of 56.7 persons ha^−1^ year^−1^ compared to 44 person’s ha^−1^ year^−1^ (2-year mean) with CT-based system (Sc1). Sc6, Sc5 and Sc4 could meet out the protein demand of 75.0, 43.2 and 41.6% more adults equivalent to 33, 19 and 18 more persons ha^−1^ year^−1^ as compared to Sc1 (Fig. 3). CA-based scenarios with mungbean could meet out the adult protein demand of 1–8 persons ha^−1^ year^−1^ more compared to without mungbean integration. Rice based systems could meet out the adult protein demand by 7.5% (3 persons ha^−1^ year^−1^) compared to Sc1. System adult`s protein, carbohydrate and fat demands were found significantly (*p* ≤ 0.05) influenced by different crops and their management practices over the years (Fig. 3). Adult`s carbohydrate demand generally followed the trend observed for carbohydrate yield. Among all scenarios, Sc5 could meet out the highest adult carbohydrate demand of 109 persons ha^−1^ year^−1^ compared to 86 persons ha^−1^ year^−1^ with Sc1 (2 years mean). Scenario 5, Sc2 and Sc3 could meet out the carbohydrate demand by 26.7, 4.7 and 2.3% more adults compared to Sc1, respectively. Among the CA-based scenarios, Sc4 could meet out the highest adult fat demand of 118 persons ha^−1^ year^−1^ compared to 12 persons ha^−1^ year^−1^ with Sc1 (2 years’ mean). Sc6 and Sc5 were also able to meet out the adult fat demand of 49 and 33 more persons ha^−1^ year^−1^ compared to Sc1 (Fig. 3).

### Economic nutrient efficiency

The 2 years results showed that system level economic efficiency of protein, carbohydrate and fat varied from 1.68–2.68, 0.26–0.53 and 2.72–18.71 USD kg^−1^, respectively, under different scenarios (Fig. 3). In term of economic efficiency of protein, Sc6 was found the most efficient (29.0% higher than Sc1) economic scenario among the all scenarios, and was able to produce 1 kg protein from 1.68 USD compared to 2.37 USD with Sc1. Sc1 required 40.9% higher money to produce the same protein yield as was with Sc6 (1.68 USD kg^−1^). The rest of the scenarios required more money to produce the protein as compared to Sc1. Rice based CA scenarios (Sc2 and Sc3) required almost similar money to get one unit of carbohydrate as was with Sc1. While other maize (Sc4 and Sc5), soybean (Sc6) and pigeon pea (Sc7) based systems required 56.3, 105.8 and 92.2%, respectively, more money to produce carbohydrate similar to Sc1 (Fig. 3). In terms of fat efficiency Sc4, Sc5 and Sc6 were found as the most efficient systems and required 83.1, 56.3, 74.8%, less money respectively, to get one unit of fat compared to Sc1 (16.05 USD kg^−1^).

### Protein water productivity

**Figure 4:**
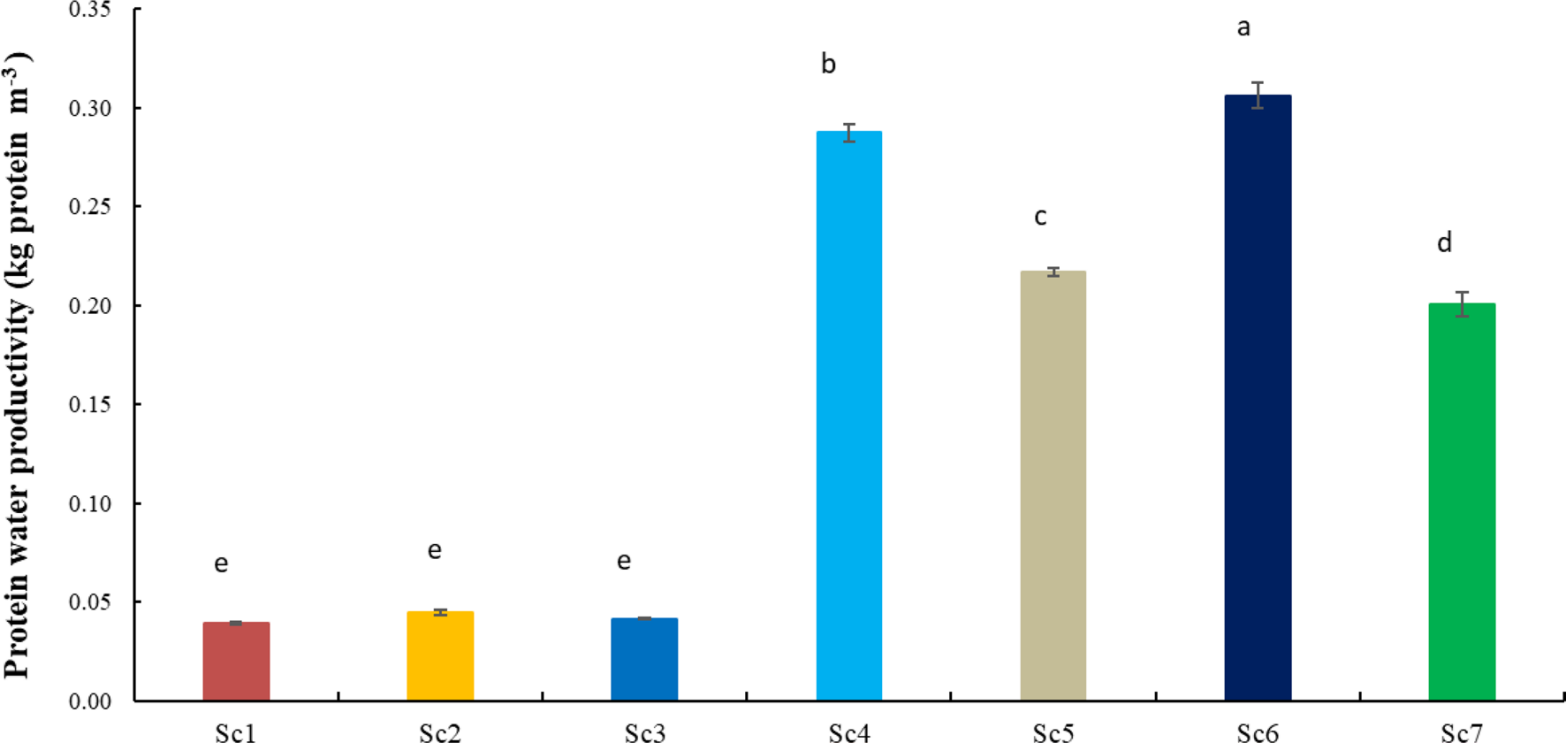
Protein water productivity (kg protein m^−3^) as affected by different cropping systems under various management scenarios (2 years mean).

The 2 years results showed that system level protein water productivity varied from 0.04 to 0.31 under different scenarios (Fig. 4). Sc6 was found the most efficient (eight times higher than Sc1) in term of protein water productivity compared to other scenarios. Similarly, Rice based CA scenarios (Sc2 and Sc3) required an almost similar amount of irrigation water to get an equal amount of protein yield as Sc1. While other maize (Sc4 and Sc5), soybean (Sc6) and pigeon pea (Sc7) based systems required 544.8, 683.4 and 412.8% less irrigation water to produce an equal protein yield as Sc1, respectively (Fig. 4).

## Discussion

### Crop and system productivity

Diversification of conventional tillage (CT)-based rice–wheat (RW) system with other remunerative crops like maize and mustard with CA-based management practices improved the systems’ productivity^3^. The rice grain yield and its system equivalent (Rice Equivalent; RE) was not much affected with different tillage (CT *vs* ZT) and crop establishment (transplanted and DSR) method in the present study period. These results are in close conformity with the earlier workers^1,2,19^. Higher maize yields on PBs might be due to the compound effects of better crop establishment method, optimum plant density, better water regimes^1,21^, less biotic and abiotic stress, active soil aeration, lesser weed population, improved soil physical health^21,22^ and improved nutrient use efficiency^10^ compared to CT-based systems. Furthermore, CA-based permanent beds moderate soil temperature and moisture creating favourable conditions by residue mulch and efficient use of irrigation water and nutrients^1,23^. In both the *kharif* seasons, PBs also reduced climatic risks from, excess rainfall, dry spell, less crops logging and lodging, resulting in luxury crop growth of soybean, pigeon pea, mustard, mungbean and maize^3,4,21^.

Higher (0.9–11.3%) wheat yields under ZT-based scenarios (Sc2–Sc7) are likely due to the implementation of portfolios of CSA practices in the right time (early sowing) right method (zero tillage), right amount (fertilizer and water) at right place (crop root zone), which brought a negative effect on weed population and a positive one on soil physico-chemical and biological properties^23,24^. In the IGP region, many researchers showed that growing rice without puddling (with aerobic methods) has positive effects on the next succeeding crops by avoiding soil compaction^2,25,26^. In the Western IGP, zero tillage offers early wheat seeding by about 2 weeks which along with residue mulch is attributable to better temperature modulation and crop protection from heat stress during wheat reproductive stage^27^. The present findings of higher wheat yield under PB planting in second year could be due to the compound effects of portfolios of CSA practices. In wheat and mustard, crop residue mulch in PBs provides favourable conditions for crop growth and yields and this is consistent with earlier observations^23,28^.

The highest system grain yield in Sc4 and Sc5 was because of the combined effect of system`s higher yield supplemented with additional yield from summer mungbean^19,25^. The yield of mungbean was highest in Sc4 in both the years, as mungbean crop gets larger window due to shorter duration of winter crop . The 3 crops in rotation (maize-mustard-mungbean) under Sc4 are so optimized that those get optimal time for maturity. Higher system yield on PBs was mainly due to residue mulch and efficient use of limited water during the dry season (summer and winter) and drainage of excess water during the rainy season leading to the higher productivity of crops in the respective season^21^. The productivity of CT-based RW system was poor mainly due to poor performance of both the crops^21,24^. However, under CA-based systems, pigeon pea-wheat system was poor mainly due to poor performance of the pigeon pea crop. The lower yield of pigeon pea in the second year was also associated with rains during flowering time which led to overgrowth of the crop and less fruit/pod setting.

### Economic profitability

Lower production cost and higher crop yields gained in CA-based systems compared with CT-based systems contributed towards the higher net returns (Table 1) in all the scenarios during both the years. Consistent with our earlier studies in the same ecology^7,19,24^, we found that the adoption of ZT reduced the production cost by 79–85% compared to conventional tillage (CT) and manual transplanting (in rice)/broadcasting (in wheat). Under PBs, the higher net income was due to less cost of cultivation in tillage and irrigation and higher grain yield of crops. In first year, very less yield of mungbean was recorded, however, during second year, no grain harvest was recorded because of less window between wheat harvesting and DSR sowing in Sc3 and that resulted in to negative (-64 USD ha^−1^) returns on two years mean basis. Mungbean integration in maize-mustard and pigeon pea-wheat improved the net returns by ~ 30% compared to other cropping systems. This crop provides a better window for mungbean cultivation^23^. Higher net return under maize, soybean and pigeon pea based systems under CA-based management system was due to cumulative effect of higher or at par yields, less cost of cultivation (in tillage, irrigation and fertilizer), and higher minimum support price (MSP). In PBs, lesser water and labour demand reduced the input costs to a greater extent compared to maize based CT scenarios^16,23^. Sustainable intensification of the CA-based MW system through mungbean integration maximized the net income, which was higher by USD 451 ha^−1^ compared to CT-based scenario (Sc1) and was mainly due to additional income generated from mungbean (Table 1). Our findings are consistent with the earlier studies by Jat et al*.*^23^ and Pooniya et al*.*^29^ who also registered higher net returns with PBs compared to flat system.

### Irrigation water use and water productivity

Diversification of conventional tillage (CT)-based rice–wheat (RW) system with other remunerative crops like maize, soybean, pigeon pea and mustard with CA-based management practices saved the systems’ irrigation water use and improved the water productivity (Fig. 2). The lower irrigation water use in direct seeded rice (DSR) scenarios (Sc2-Sc3) was mainly due to avoidance of puddling which requires water equivalents to 3–4 irrigations and in combination with crop residues retention that probably minimized the evaporation loss from the soil surface^2,26^. Replacement of rice from the rice–wheat system using other crops like maize, soybean, pigeon pea required only 5–10% of total irrigation water used by the rice–wheat system. This might be due to the lower water requirement of these crops. Diversified systems layered with water smart (furrow irrigation, tensiometer based irrigation, use of ICTs for precise weather information) and carbon smart (reside retention, zero-tillage, mungbean integration) agricultural practices resulted in more lower water use and higher water productivity compared to the traditional method of irrigation (border irrigation based on crop morphology). These results are in close conformity with those reported by Jat et al*.*^19,23^ and Pooniya et al*.*^29^ under different cropping systems. In wheat and mustard, PBs reduced irrigation water by ~ 33% (2-years’ mean) compared to Sc1 (Fig. 2). The highest irrigation water productivity (WP_I_) was recorded with CA-based maize-mustard-mungbean (Sc4) system (~ 3.18 kg grain m^−3^) followed by CA-based maize-wheat-mungbean (Sc5) system (~ 2.39 kg grain m^−3^) compared to CT-based Sc1 (0.54 kg grain m^−3^). This was mainly due to less irrigation water used coupled with higher grain yields of respective crops under the different cropping system. Similar results of higher WP_I_ in CA-based Sc4 and Sc5 in the IGP of India were also recorded by many researchers^1,19^. Higher values of WP_I_ in the MW system on PBs compared to flat planting were also reported by Jat et al*.*^28^.

### Protein yield and adult demand equivalent

Presently, RW system in the western IGP is facing challenges of exaggerating decline in input use efficiencies and, soil and environmental quality. Therefore, diversification of cereal crops with pulses and oilseeds is required to achieve nutritional security in the region^30^. CA-based crop diversification in this study showed a potential to combat the identical challenges of declining value of natural resources and import of protein and fat (oil and pulses). Crop diversification provides a diversity of diet (protein, carbohydrate and fat) and improves their yield, income and nutritional security (Table 2). Higher protein yield was recorded in all the scenarios where pulse crops (soybean, pigeon pea and mungbean) were included in the system. Soybean based CA scenario (Sc6) produced higher protein yield because soybean and mungbean contain ~ 40–42 and 20–25% protein, respectively, that resulted in higher protein yield^31^. Higher protein yield with mungbean integration in the RW system was also reported by Jat et al*.*^19^ and Parihar et al*.*^16^. CA-based management systems improved the protein yield by providing window for mungbean cultivation in between wheat harvest and rice sowing compared to CT based system^7^. Therefore, Sc6 could meet out the highest adult protein demand of 48 person’s ha^−1^ year^−1^ compared to 19 person’s ha^−1^ year^−1^ with Sc1 (2 years mean). This was mainly due to high protein (40–42%) and oil content (20–22%) as well as more yield with best management practices, soybean crop is among the best sources of plant-based protein^32^. Therefore, this is considered to be an important food product for reducing malnutrition^33^. Maize based scenarios (Sc4 and Sc5) also produced the higher protein yield because mustard and mungbean grain contain 30–35 and 20–50% protein on dry matter basis, respectively. Therefore, these scenarios could meet the adult protein demand of 16 and 19 person’s ha^−1^ year^−1^ extra compared to farmers’ practice (Sc1). The Sc4 was more economically efficient in terms of protein because it contained lower protein content and higher net returns as compared to CT based RW system.

### Carbohydrate yield and adult demand equivalent

Carbohydrate yield was influenced by the crop yields and their carbohydrate content in the grains. The carbohydrate content varies from 70 to 78% in rice, maize and wheat and influenced to some extent by the management practices^34^. However, in pulses and oilseeds, the carbohydrate is almost half of the content found in cereal crops and varies from 30 to 40% only. Maize based scenario (Sc5) produced highest carbohydrate yield compared to all other scenarios. The highest carbohydrate yield was associated with the higher grain yield of the maize and wheat crop in the respective scenarios, as, maize grains contain 75–78% and wheat 70–73% carbohydrate^31^. The higher carbohydrate yield under CA-based maize-wheat-mungbean system could be associated with higher grain yield and supplemented with the commendatory soil temperature/moisture conditions, improved soil properties, better water and nutrient uses besides, amalgamating the effects of the residue retention^16^. Application of best crop management practices improved the nutritional quality of the crops by increasing the availability of nutrients from the surface layer under CA-based cropping systems^10^. Higher carbohydrate yield of maize-wheat-mungbean system (Sc5) could meet out 23 person’s ha^−1^ year^−1^ more adult protein demand compared to Sc1.The Sc6 and Sc7 were more economically efficient in terms of carbohydrate because it contained lower carbohydrate content and higher net returns as compared to CT based RW system.

### Fat yield and adult demand equivalent

Fat yield of different cropping systems is proportional to fat content in the grains and the crop yields. Highest fat yield was recorded under CA-based scenario (Sc4, Sc5 and Sc6) because of higher fat content in associated crops of the scenarios. The fat yield under the different scenarios is directly proportional to the fat content in individual crop and their actual yields. Higher yield with Sc4 was owing to integration of mustard instead of wheat that contained higher fat percentage (28–32%). Soybean contained 18–20% fat content that resulted in second highest fat yield^35^. In maize, rice and wheat fat content is 4–5, 0.5–1 and 1–2%, respectively, which is very low, compared to oilseeds (mustard and soybean)^36^. Additional fat yield under CA-based Sc4, Sc6 and Sc5 could meet the adult fat demand of 99, 32 and 49 person’s ha^−1^ year^−1^, respectively, compared to 13 persons ha^−1^ year^−1^ with Sc1. This might be due to the higher fat content in the respective crops. The Sc2, Sc3 and Sc7 were more economically efficient in terms of fat because it contained lower fat content and higher net returns as compared to CT based RW system.

### Protein water productivity

Diversification of CT-based rice–wheat system with other rotations involving cropss like maize, soybean, pigeon pea, mustard and mungbean with CA-based management practices saved the irrigation water while increased the protein yield per unit of water use (protein water productivity) (Fig. 4). The highest protein water productivity was recorded with CA-based soybean-wheat-mungbean (Sc6) system (~ 0.31 kg protein m^−3^) followed by CA-based maize-mustard-mungbean (Sc4) system (~ 0.29 kg protein m^−3^) and lowest under CT-based Sc1 (0.04 kg protein m^−3^). This was mainly due to less irrigation water used coupled with higher nutrient yield of respective crops under the different cropping system.

## Conclusion

Agroecological approaches such as Conservation Agriculture (CA)-based cropping system diversification might help addressing the critical issues in farming and increase farm income while ensuring sustainable and healthy food and ecological security in smallholder systems of South Asia. Our study demonstrated that diversified cropping systems with CA-based management optimization increased the system productivity (+ 16%), profitability (+ 27%), protein yield (+ 30%) and protein water productivity (+ 368%) compared to CT-based rice–wheat system. Maize-wheat-mungbean on permanent beds was found as the most efficient production system, which resulted in 32.3% more grains, 57.4% higher economic profitability along with 43.8, 27.5 and 259.8% higher protein, carbohydrate and fat yields, respectively, compared to farmers’ business as usual practice (CT- based RW system). Our study, therefore, demonstrated that, CA-based, maize-mustard-mungbean, maize-wheat-mungbean and soybean-wheat-mungbean rotation are potential scalable alternatives to RW system to address the critical challenges of deteriorating natural resources and import dependence on protein and fat in the form of oil and pulses to contribute to food and nutritional security on a sustainable basis.

## Method and materials

### Experimental site characteristics

The present study was carried out for 2 years (2018–2020) at experimental farm of ICAR-Central Soil Salinity Research Institute, Karnal (29° 42ʺ20.7ʹ N latitude, 76° 57ʺ19.79ʹ E longitude). The region has a semi-arid condition with sub-tropical climate characterized by wet summers and dry winters and having three distinct seasons i.e. *Kharif* (July-Oct), *Rabi* (Nov-Mar) and *Zaid* (April–June). The cyclonic rains are received through south-west monsoon and the region receives an average annual rainfall of 670 mm, 70–80% of which occurs from June to Sep (monsoon season). The soil of the experimental field was silty loam in texture, low in organic carbon (0.48%) with slightly alkaline pH (8.13).

### Experimental details and description of scenarios

**Figure 5:**
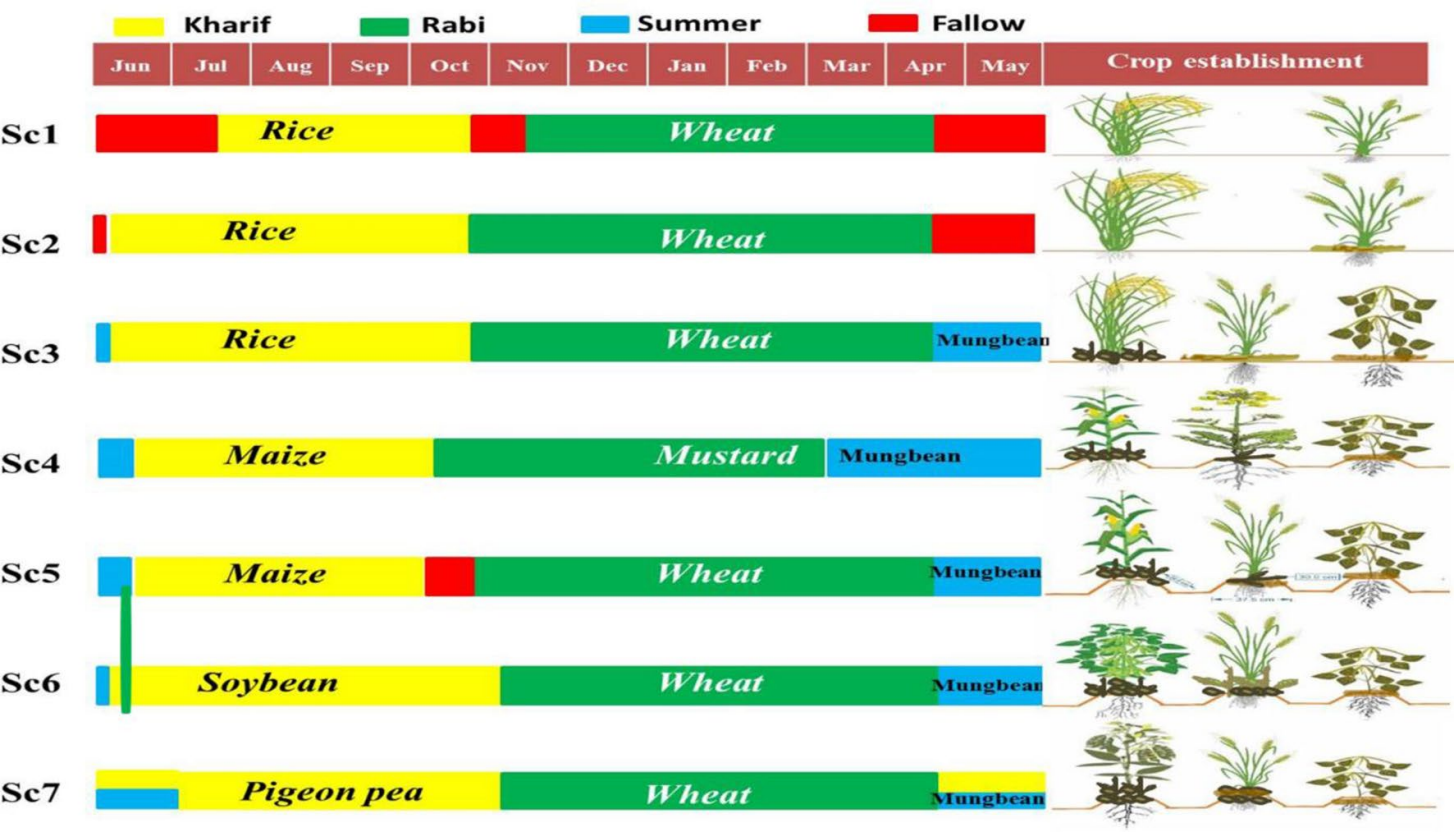
Schematic diagram of different crops and cropping sequence under different crop establishment methods**.**

In this study, a portfolio of management practices has been evaluated under different crops and cropping systems (Fig. 5). Seven combination of treatments with different crop rotations and associated management practices referred as scenarios (Sc) were evaluated as per the prevailing condition in the western Indo-Gangetic plains. Seven scenarios with the layering of different agronomic management practices were as follows: Sc1 (FP; farmers’ practice)-puddled transplanted rice (PTR) followed by (fb) conventional tillage (CT) wheat without residue(-R); Sc2-CT direct seeded rice (CTDSR) fb zero tillage wheat (ZTW) with residue(+ R); Sc3- ZTDSR fb ZTW fb ZT mungbean(+ R); Sc4-ZT raised beds (PBs; permanent beds) based maize-mustard-mungbean system(+ R); Sc5-maize-wheat-mungbeansystem on PBs (+ R); Sc6- soybean-wheat-mungbean system on PBs (+ R); Sc7-pigeon pea-wheat-mungbean system on PBs (+ R) were tested and evaluated for productivity, profitability and nutrition. Each scenario was replicated thrice in a production scale plot (12 m × 50 m = 600 m^2^) in a randomized complete block design. All the management practices in Sc1 was as per the current farmers’ practice in the region, whereas Sc2, Sc3, Sc4, Sc5, Sc6 and Sc7 were based on conservation agriculture (CA) based management practices. The description of different scenarios and their management practices are presented in Tables 4 and 5. Handling of plants was carried out in accordance with relevant guidelines and regulations of CCSHAU, Hisar and ICAR-CSSRI, Karnal. Seeds of all different crop varieties taken in this study are commercially available in India.

**Table 4:** Crop rotation, tillage and crop establishment method, residue management and water management protocols under different scenarios.

Scenarios	Crop rotations	Tillage	Crop establishment method	Residue management	Water management
Sc1	Rice–wheat-fallow	CT-CT	Rice: Puddled transplanted rice with random geometry Wheat: Conventional till (CT) with broadcast seeding	All crop residues removed	Border irrigation
Sc2	Rice–wheat-fallow	CT-ZT	Rice: CT direct seeded rice (DSR) with row geometry Wheat: Zero tillage (ZT) wheat with row geometry	Full (100%) rice residue retained and anchored (25–30%) wheat residue incorporated	Same as Sc1
Sc3	Rice–wheat-mungbean	ZT-ZT-ZT under flats	All crops under ZT with row geometry	Full rice and mungbean, and anchored wheat residue retained	Same as Sc1
Sc4	Maize-mustard-mungbean	ZT-ZT-ZT under permanent beds (PBs)	Same as Sc3	Anchored residue of maize(60–70%) and mustard (30–40%), and full mungbean residue retained	Furrow irrigation
Sc5	Maize-wheat-mungbean	Same as Sc4	Same as Sc3	Anchored residue of maize, and wheat, and full mungbean residue retained	Same as Sc4
Sc6	Soybean-wheat-mungbean	Same as Sc4	Same as Sc3	Anchored residue of soybean (25–30%), wheat and full mungbean residue retained	Same as Sc4
Sc7	Pigeon pea-wheat-mungbean	Same as Sc4	Same as Sc3	Anchored residue of pigeon pea (20–25%), wheat and full mungbean residue retained	Same as Sc4

*CT* conventional tillage, *ZT* zero tillage, *PBs* permanent beds, *DSR* direct seeded rice.

**Table 5:** Crop management practices for crops and cropping systems under different management scenarios.

Scenarios^a^/ Management practices	Scenario 1	Scenario 2	Scenario 3	Scenario 4	Scenario 5	Scenario 6	Scenario 7
Field preparation	Rice- 2 pass of harrow, 1 pass of rotavator, 2 pass of puddle harrow followed by (fb) planking Wheat- 2 pass of harrow and rotavator each fb planking	Rice-1 pass of harrow, 1 pass of cultivator fb planking Wheat- Zero tillage	Zero tillage (ZT) on flats	ZT on permanent beds (Pbs)	ZT on permanent beds (Pbs)	ZT on permanent beds (Pbs)	ZT on permanent beds (Pbs)
Seed rate (kg ha^-1^)	Rice- 12.5 Wheat- 100	Rice- 20 Wheat- 100	Rice- 20 Wheat-100 Mungbean- 20	Maize- 20 Mustard- 05 Mungbean- 20	Maize- 20 Wheat- 100 Mungbean- 20	Soybean- 20 Wheat- 100 Mungbean- 20	Pigeon pea- 15 Wheat- 100 Mungbean- 20
Sowing method	Manual transplanting of rice and broadcasting of wheat	Rice seeding with multi-crop planter and wheat seeding with Happy Seeder machine	Seeding with Happy Seeder machine	Seeding with double disc planter	Seeding with double disc planter	Seeding with double disc planter	Seeding with double disc planter
Crop geometry (cm)	Random geometry	22.5—22.5	22.5—22.5—45	67.5—33.7 – 33.7	67.5—33.7 – 33.7	33.7—33.7 – 33.7	67.5—33.7 – 33.7
Fertilizers (N:P:K) in kg ha^-1^	Rice- 195:57.5:00 Wheat- 166:57.5:00 ZnSO_4_ @25 kg ha^-1^	Rice- 150:60:60 Wheat- 150:60:60; ZnSO_4_ @25 kg ha^-1^	Rice- 150:60:60 ZnSO_4_ @25 kg ha^−1^ Wheat- 150:60:60 Mungbean- 20:40:00	Maize- 150:60:60 Mustard- 80:30:20 Mungbean- 20:40:00	Maize- 150:60:60 Wheat- 150:60:60 Mungbean- 20:40:00	Soybean-25:80:00 Wheat- 150:60:60 Mungbean- 20:40:00	Pigeon pea-20:40:00 Wheat- 150:60:60 Mungbean- 20:40:00
Water management (no. of irrigations)	Rice- Continuous flooding of 5–6 cm depth for 50–60 days after transplanting fb irrigation applied at alternate wetting and drying (30–35 irrigations) Wheat- 4–6	Rice- Soil was kept wet up to 20 days after sowing fb irrigation applied at hair-line cracks (30–40 irrigations) Wheat- 4–6	Same as scenario 2 Wheat- 4–6 Mungbean- 2–3	Maize- 4–5 irrigations Mustard- 4–6 Mungbean- 2–3	Maize- 4–5 Wheat- 4–6 Mungbean- 2–3	Soybean- 2–4 Wheat- 4–6 Mungbean- 2–3	Pigeon pea- 4–5 Wheat- 4–6 Mungbean- 2–3
Crop variety	Rice- Arize 6129 Wheat- HD 2967	Rice- Arize 6129 Wheat- HD 2967	Rice- Arize 6129 Wheat- HD 2967 Mungbean- MH 421	Maize- CP 858 Mustard- CS 58 Mungbean- MH 421	Maize- CP 858 Wheat- HD 2967 Mungbean- MH 421	Soybean- SL 958 Wheat- HD 2967 Mungbean- MH 421	Pigeon pea- PADT 16 Wheat- HD 2967 Mungbean- MH 421

### Crop residue management under different scenarios

In farmers’ practice (Sc1), all the crop residues were removed from each crop. In Sc2, all rice residues were retained but anchored wheat residues were incorporated before conventional-till direct seeded rice (DSR) sowing. However, in Sc3 all rice residue and anchored wheat residue were retained on the soil surface. In Sc 4, and 5, partial (60–65%; ~ 150 cm from soil surface or just above the cob) maize residues and anchored wheat stubbles (25–30%; ~ 15 cm from the surface) were retained at the soil surface. In Sc 6 and 7, soybean (~ 25–30%) and pigeon pea (~ 20–25%) residues and anchored wheat residue were retained on the soil. All mungbean residues were retained at the soil surface in respective scenarios.

### Fertilizer and weed management

Protocols related to fertilizer management in each crops under different scenario are presented in Table 5. All the crops were fertilized with recommended dose of fertilizers (RDF) over both the years. From Sc1 to Sc3, 25 kg ZnSO_4_ ha^−1^ was applied to rice crop only. Whole P, K and Zn were applied as basal at sowing/transplanting time, while remaining N was top dressed as urea in two to three equal splits depending on the sensitive stages of crops. Full dose of NPK was applied at the time of sowing in leguminous crops (soybean, pigeon pea, and mungbean).

A pre-plant application of glyphosate 1.25 l a.i. per hectare was applied to manage the weeds in permanent beds and zero-till plots. The weeds in the experimental plots were controlled through pre-emergence and post-emergence herbicides as per the standard recommendation. The herbicides used for weed control in DSR (CT/ZT) were: pendimethalin (1000 g a.i. ha^−1^) as pre-emergence followed by Bispyribac Sodium + Pyrazosulfuron ethyl (8–10 g + 6 g a.i. ha^−1^, respectively) at 20–25 days after sowing (DAS) to control all grassy and broad leaf weeds and sedges. In maize, atrazine (1000 g a.i. ha^−1^) followed by Laudis Tembotrione 42% SC (90 g a.i. ha^−1^) were used as pre- and post-emergence herbicides depending on the intensity and diversity of weed species, respectively. In soybean and pigeon pea, pre- (2 DAS) and post-emergence (40 DAS) of pendimethalin (1500 ml ha^−1^) or post-emergence spray of Imazethapyr and Quizalofop ethyl (750 ml ha^−1^) were applied at 15–20 DAS, respectively. In wheat, tank mix solution of Pinoxaden 5% EC (50 g a.i. ha^−1^) or Clodinafop ethyl + Metsulfuron (60 + 4 g a.i. ha^−1^) was applied at 30–35 DAS to control all types of weeds.

### Irrigation water management

A polyvinyl chloride (PVC) pipeline was installed in a 60 cm deep trench with an outlet for each plot separately. On-line water meter (Woltman helical turbine) was fitted for irrigation water measurement. Water meter readings were recorded for each irrigation to calculate the amount of irrigation water applied per plot. The amount of irrigation water applied (mm ha^−1^) and water productivity for irrigation (WP_I_) were calculated using Eqs. (1–3).

1 $${\text{Volume}}\;{\text{of}}\;{\text{irrigation}}\;{\text{water}}\;({\text{kilolitre}}\;{\text{ha}}^{{ - {1}}} ) = \left\{ {\left( {{\text{final}}\;{\text{water}}\;{\text{meter}}\;{\text{reading}} - {\text{initial}}\;{\text{water}}\;{\text{meter}}\;{\text{reading}}} \right)/{\text{plot}}\;{\text{area}}\;{\text{in}}\;{\text{m}}^{{2}} } \right\}*{1}0000$$

2 $${\text{Irrigation}}\;{\text{water}}\;({\text{mm ha}}^{{ - {1}}} ) = {\text{volume}}\;{\text{ of}}\;{\text{ irrigation}}\;{\text{water}}\; \, ({\text{kilolitre}}\;{\text{ha}}^{{ - {1}}} )/{1}0$$

3 $${\text{Irrigation}}\;{\text{water}}\;{\text{productivity}} = {\text{grain}}\;{\text{yield}}\;\left( {{\text{kg}}\;{\text{ha}}^{{ - {1}}} } \right)/{\text{irrigation}}\;{\text{water}}\;{\text{used}}\;\left( {{\text{mm}}\;{\text{ha}}^{{ - {1}}} } \right)$$

In both border and furrow irrigation systems, number of irrigations varied from 2 to 6 except rice crop. Water management protocols for each scenario are presented in Table 5.

### Crop yield and net returns

Rice and wheat crops were harvested and threshed either manually or using a combine harvester at a height of 30 cm above ground level except in Sc1, which was harvested at ground level; whereas, maize, soybean, pigeon pea and mustard crops were harvested and threshed manually. At maturity, the grain and straw yields of both wheat and rice were determined on a total area of 100 m^2^ by sampling from four locations of 25 m^2^ each. Grain and straw yields of maize, soybean, pigeon pea, mustard and mungbean were estimated by harvesting a total area of 108 m^2^ from each plot by sampling from four locations of 27 m^2^ each. For mungbean yield estimation, the entire plot was harvested and weighed. To compare the productivity of different crops and total system productivity of the different scenarios, the yield of non-rice crops (wheat, maize, soybean, pigeon pea, mustard and mungbean) was converted into rice equivalent yield (REY) (Mg ha^−1^) and calculated using the Eq. (4).

4 $${\text{Rice}}\;{\text{equivalent}}\;{\text{yield}} = {\text{grain}}\;{\text{yield}}\;{\text{of}}\;{\text{ non{-}rice}}\;{\text{crop}}\;\left( {{\text{Mg}}\;{\text{ha}}^{{ - {1}}} } \right)*{\text{MSP}}\;{\text{of}}\;{\text{non{-}rice}}\;{\text{crop}}\;\left( {{\text{USD Mg}}^{{ - {1}}} } \right)/{\text{MSP}}\;{\text{of}}\;{\text{rice}}\;\left( {{\text{USD}}\;{\text{Mg}}^{{ - {1}}} } \right)$$

where, MSP is the minimum support price (Table S1); (1 USD = 70 INR).

The data on crop management inputs such as number of tillage operations, fuel consumption, number of irrigations, herbicide, fertilizer, seed rate, labour use, pesticide application and their costs under each treatment were recorded for each crop using a standard data recording format. All these costs were summed up to calculate the total cost of production. Cost of key inputs and outputs used for economic analysis during the different years are presented in Table S1. Gross returns were obtained as per the prevailing market prices of the commodity (grain and straw/stover) over the different years. Net returns were calculated by deducting the total cost of cultivation from the gross returns.

### Analysis of nutrient quality parameters

Grain quality parameters like protein, carbohydrate and fat contents were computed from all the crops. The protein content was calculated using the nitrogen (N) content in grain and grain yield of individual crops. N content in the grain was determined as per Kjeldhal method^37^. N content in the grain varied from 1.54 to 1.59% in wheat, 1.06–1.09% in rice, 1.1–1.4% in maize, 5.5–5.9% in soybean, 3.2–3.4% in pigeon pea, 3.44–3.69% in mustard, and 3.61–3.69% in mungbean. Final value of the protein was calculated by multiplying the detected N content with a standard factor for each crop (5.95 for rice; 5.80 for wheat; 6.25 for maize; 5.71 for soybean; 6.25 for pigeon pea; 5.30 for mustard and 5.70 for mungbean) as given by Mariotti et al.^38^ Calorimetric method/phenol- sulphuric acid method was used to determine carbohydrate concentration in the grains^39^. The sulphuric acid causes all non-reducing sugars to be converted to reducing sugars, so that this method determines the total sugars present. Fat content in the grains was determined with the Soxtec-Avanti 2050 total fat system (Foss Co., Denmark) method^40^. The yield efficiency of protein, carbohydrate and fat was calculated based on the annual adult protein, carbohydrate and fat demand equivalent based on the 58, 30 and 275 g person^−1^ day^−1^, respectively, as per the recommendations of the Indian council of medical research^41^.

### Economic-efficiency (EE) of nutrients

Economic-efficiency (EE) is an index aimed at de-coupling resource use and output produced from economic activity and the economic-efficiency indicator is defined as a ratio between economic value added and a output produced (protein, carbohydrate and fat). In this study, economic-efficiency of protein, carbohydrate and fat yield was calculated using the Eqs. (5–7) as per Kakraliya et al*.*^42^

5 $${\text{Economic}}\;{\text{efficiency}}\;{\text{of}}\;{\text{protein}}\;\left( {{\text{USD}}\;{\text{kg}}^{{ - {1}}} } \right) = {\text{economic}}\,{\text{return}}\;\left( {{\text{USD}}\;{\text{ha}}^{{ - {1}}} } \right)/{\text{protein}}\;{\text{yield}}\;\left( {{\text{kg}}\;{\text{ha}}^{{ - {1}}} } \right)$$

6 $${\text{Economic}}\;{\text{efficiency}}\;{\text{of}}\;{\text{carbohydrate}}\;\left( {{\text{USD kg}}^{{ - {1}}} } \right) = {\text{economic}}\;{\text{return}}\;\left( {{\text{USD}}\;{\text{ha}}^{{ - {1}}} } \right)/{\text{carbohydrate}}\;{\text{yield}}\;\left( {{\text{kg}}\;{\text{ha}}^{{ - {1}}} } \right)$$

7 $${\text{Economic}}\;{\text{efficiency}}\;{\text{of}}\;{\text{fat}}\;\left( {{\text{USD}}\;{\text{kg}}^{{ - {1}}} } \right) = {\text{economic}}\;{\text{return}}\;\left( {{\text{USD}}\;{\text{ha}}^{{ - {1}}} } \right)/{\text{fat}}\;{\text{yield}}\;\left( {{\text{kg}}\;{\text{ha}}^{{ - {1}}} } \right)$$

### Protein water productivity

The protein water productivity is defined as amount of water required (evaporated or used directly) to produce unit protein yield. Protein water productivity was calculated using the Eq. (8)

8 $${\text{Protein}}\;{\text{water}}\;{\text{productivity}} = {\text{protein}}\;{\text{yield}}\;\left( {{\text{kg}}\;{\text{ha}}^{{ - {1}}} } \right)/{\text{irrigation}}\;{\text{water}}\;{\text{used}}\;\left( {{\text{mm}}\;{\text{ha}}^{{ - {1}}} } \right)$$

### Statistical analysis

The data recorded for different crop parameters were analysed using analysis of variance (ANOVA) technique^43^ for randomized block design using SAS 9.1 software^44^. The treatment means were compared using Turkey’s honestly significant difference (HSD at 5% level of significance).

## Supplementary Information

Supplementary Information.

## Data Availability

The datasets used and /or analysed during the current study available from the corresponding author on reasonable request.
