# Supplementary material for: Scalable diversification options delivers sustainable and nutritious food in Indo-Gangetic plains
Source: Sci Rep. 2022 Aug 23;12:14371. doi: 10.1038/s41598-022-18156-1 (PMC9399183; doi:10.1038/s41598-022-18156-1)
Supplement: Supplementary file 1 — Supplementary Information. [file 41598_2022_18156_MOESM1_ESM.docx]

**Table S1.** Cost of key inputs and outputs used for economic analysis during project period

| Item/Commodity | Units | Cost (INR) | |
| --- | --- | --- | --- |
|  |  | 2081-19 | 2019-20 |
| Rice/maize/soybean/pigeon pea/wheat/mustard/mungbean | MSP(USD) kg^-1^ grains | 0.25/0.24/0.49/0.81/0.26/0.60/1.01 | 0.26/0.25/0.53/0.83/0.28/0.63/1.03 |
| Wheat | USD kg^-1^ straw | 0.04 | 0.04 |
| Rice/maize/soybean/pigeon pea/wheat/mustard/mungbean | USD kg^-1^ seeds | 3.57/3.57/0.57/2.14/0.64/1.71/1.43 | 3.57/3.57/0.57/2.14/0.64/1.71/1.43 |
| Urea(kg^-1^) | USD kg^-1^ | 0.08 | 0.08 |
| Di-ammonium phosphate (DAP) (kg^-1^) | USD kg^-1^ | 0.37 | 0.37 |
| Muriate of potash (MOP) (kg^-1^) | USD kg^-1^ | 0.24 | 0.24 |
| NPK complex (kg^-1^) | USD kg^-1^ | 0.32 | 0.32 |
| Zinc sulphate (kg^-1^) | USD kg^-1^ | 0.51 | 0.51 |
| Harrowing (ha^-1^) | USD ha^-1^ | 17.86 | 17.86 |
| Cultivator (ha^-1^) | USD ha^-1^ | 17.86 | 17.86 |
| Planking (ha^-1^) | USD ha^-1^ | 10.71 | 10.71 |
| Puddler (ha^-1^) | USD ha^-1^ | 21.43 | 21.43 |
| Rotavator (ha^-1^) | USD ha^-1^ | 35.71 | 35.71 |
| Happy Seeder (ha^-1^) | USD ha^-1^ | 42.86 | 42.86 |
| Double disk bed planter (ha^-1^) | USD ha^-1^ | 17.86 | 17.86 |
| Seed drill (ha^-1^) | USD ha^-1^ | 17.86 | 17.86 |
| Wages rate (person^-1^ day ^-1^) | USD day^-1^ | 5.43 | 5.43 |
| USD ($) to INR Conversation rate |  | 70 | 70 |

*Where:* MSP is Minimum support price, USD is US dollar

**Table S2.** Irrigation water and water productivity of different crops and cropping systems as affected by different management practices during the year 2018-19 and 2019-20

| Scenarios^a^ | Irrigation water (mm ha^-1^) | | | Water productivity (kg grain m^-3^) | | |
| --- | --- | --- | --- | --- | --- | --- |
|  | Rice/maize/ soybean/ pigeon pea | Wheat/ mustard | System | Rice/maize/ soybean/ pigeon pea | Wheat/ mustard | System |
| *Year 2018-19* | | | | | |  |
| Sc1 | 1445^A^ | 555^A^ | 2000^B^ | 0.40^E^ | 1.18^D^ | 0.63^D^ |
| Sc2 | 1336^C^ | 509^A^ | 1845^C^ | 0.45^E^ | 1.38^CD^ | 0.72^D^ |
| Sc3 | 1409^B^ | 491^C^ | 2074^A^ | 0.40^E^ | 1.45^BC^ | 0.66^D^ |
| Sc4 | 109^D^ | 136^F^ | 423^F^ | 6.45^B^ | 2.00^A^ | 3.79^A^ |
| Sc5 | 112^D^ | 414^D^ | 648^D^ | 6.99^A^ | 1.63^B^ | 2.37^B^ |
| Sc6 | 110^D^ | 409^D^ | 656^D^ | 2.61^C^ | 1.40^CD^ | 1.85^C^ |
| Sc7 | 96^E^ | 391^E^ | 618^E^ | 1.30^D^ | 1.48^BC^ | 2.03^C^ |
| *Year 2019-20* | | | | | |  |
| Sc1 | 2782^A^ | 245^A^ | 3027^A^ | 0.21^D^ | 1.98^D^ | 0.37^D^ |
| Sc2 | 2709^C^ | 240^AB^ | 2949^C^ | 0.21^D^ | 2.38^C^ | 0.40^D^ |
| Sc3 | 2737^B^ | 241^AB^ | 2978^B^ | 0.20^D^ | 2.28^CD^ | 0.38^D^ |
| Sc4 | 273^E^ | 73^D^ | 491^E^ | 3.49^A^ | 2.80^B^ | 3.51^A^ |
| Sc5 | 282^D^ | 227^B^ | 582^D^ | 3.53^A^ | 2.42^BC^ | 2.74^C^ |
| Sc6 | 200^F^ | 155^C^ | 436^F^ | 1.51^B^ | 3.88^A^ | 2.97^B^ |
| Sc7 | 100^G^ | 145^C^ | 391^G^ | 0.75^C^ | 3.94^A^ | 2.90^B^ |

^a^Refer Table 4 for description of scenarios

^b^Means followed by a similar uppercase letter within a column are not significantly different at 0.05 level of probability using Tukey’s HSD test.
